# Oxidative Stress Induces Skin Pigmentation in Melasma by Inhibiting Hedgehog Signaling

**DOI:** 10.3390/antiox12111969

**Published:** 2023-11-06

**Authors:** Nan-Hyung Kim, Ai-Young Lee

**Affiliations:** Department of Dermatology, Dongguk University Ilsan Hospital, 814 Siksa-dong, Ilsandong-gu, Goyang-si 410-773, Gyeonggi-do, Republic of Korea

**Keywords:** oxidative stress, primary cilia, hedgehog signaling, keratinocyte differentiation, skin hyperpigmentation

## Abstract

There is growing evidence that oxidative stress plays a role in melasma and disrupts primary cilia formation. Additionally, primary cilia have been suggested to have an inhibitory role in melanogenesis. This study examined the potential link between oxidative stress, skin hyperpigmentation, and primary cilia. We compared the expression levels of the nuclear factor E2-related factor 2 (NRF2), intraflagellar transport 88 (IFT88), and glioma-associated oncogene homologs (GLIs) in skin samples from patients with melasma, both in affected and unaffected areas. We also explored the roles of NRF2, IFT88, and GLIs in ciliogenesis and pigmentation using cultured adult human keratinocytes, with or without melanocytes. Our findings revealed decreased levels of NRF2, heme oxygenase-1, IFT88, and GLIs in lesional skin from melasma patients. The knockdown of *NRF2* resulted in reduced expressions of IFT88 and GLI1, along with fewer ciliated cells. Furthermore, *NRF2*, *IFT88*, or *GLI1* knockdown led to increased expressions in protease-activated receptor-2 (PAR2), K10, involucrin, tyrosinase, and/or melanin. These effects were reversed by the smoothened agonist 1.1. Calcium also upregulated these proteins, but not NRF2. The upregulation of involucrin and PAR2 after *NRF2* knockdown was mitigated with a calcium chelator. In summary, our study suggests that oxidative stress in NRF2-downregulated melasma keratinocytes impedes ciliogenesis and related molecular processes. This inhibition stimulates keratinocyte differentiation, resulting in melanin synthesis and melanosome transfer, ultimately leading to skin hyperpigmentation.

## 1. Introduction

Melasma, a common facial skin pigmentation disorder, poses ongoing challenges in unravelling its complex pathogenesis. While genetic background and female sex hormones are recognized contributors, chronic exposure to ultraviolet (UV) radiation is considered a key trigger [1]. UV exposure generates reactive oxygen species (ROS), resulting in oxidative stress when the body’s antioxidant defenses are overpowered [2]. Studies have pointed to the role of oxidative stress in melasma development [3,4,5,6].

The impact of oxidative stress on ciliogenesis disruption has been explored in several reports [7,8], yet there has been no skin-specific investigations. The role of primary cilia in the negative regulation of melanogenesis was investigated in a study using cultured melanocytes [9]. The factors leading to ciliogenesis inhibition and the precise mechanisms governing the interplay between primary cilia and skin pigmentation remain elusive. However, the evidence implicating oxidative stress in ciliogenesis disruption and the inhibitory role of primary cilia in melanogenesis suggest a compelling link between oxidative stress, ciliogenesis inhibition, and enhanced melanogenesis.

Primary cilia, antenna-like organelles projecting from the apical surfaces of most eukaryotic cells, serve as sensory organelles that receive and transduce environmental signals into coordinated cellular responses, influencing various cellular processes [10]. Structural alterations in cilia, such as shortening and/or loss, can compromise their signaling capabilities, resulting in defects that contribute to developmental issues, degenerative diseases, and cancer progression. The core of the primary cilium contains an axoneme composed of a ring of nine microtubule doublets that extend from the basal body at the base of the cilium, derived from the mother centriole [11]. Among the numerous genes involved in ciliogenesis and ciliary functions [12], intraflagellar transport (IFT), a bi-directional transport system, plays a critical role in elongating the cilium axoneme during ciliary assembly [13]. The hedgehog (Hh) pathway represents a central main mechanism for cilium-based signaling [14,15], requiring key proteins such as patched (PTCH), smoothened (SMO), and glioma-associated oncogene homologs (GLIs). The PTCH homolog, a transmembrane receptor for the secreted Sonic hedgehog (Shh) protein, triggers SMO homologs to release the suppressor of the fused homolog–zinc finger protein-GLI complex, enabling the GLIs’ nuclear translocation and activation of Hh target gene transcription [16,17,18].

This study aimed to explore the potential link among oxidative stress, primary cilia, and skin hyperpigmentation in melasma. While it is important to acknowledge that approximately 10% of melasma cases occur in men, making gender-based comparisons challenging, the study primarily focused on understanding the inter-individual variations in melasma pathogenesis [1]. To do so, we compared the expression levels of nuclear factor E2-related factor 2 (NRF2) and the number of primary cilia, along with the related molecule expression, including IFT88, GLIs, and PTCH homologs, between lesional and non-lesional skin specimens from the same melasma patients. Furthermore, we investigated the roles of NRF2, IFT88, and GLI1 in ciliogenesis and melanogenesis using primary cultured adult human keratinocytes and keratinocyte–melanocyte cocultures.

## 2. Materials and Methods

### 2.1. Patients

This study population comprised 16 female patients diagnosed with melasma, aged 29–62 years (mean age: 51.7 years). Ethical approval was obtained from the Institutional Review Board of the Dongguk University Ilsan Hospital, and the study adhered to the principles outlined in the Declaration of Helsinki. Written informed consent was acquired from each patient. Paired sets of hyperpigmented and adjacent normally pigmented skin specimens were retrieved through biopsy for direct comparisons. The specimens were subjected to real-time polymerase chain reaction (PCR) and immunohistochemistry.

### 2.2. Normal Human Epidermal Cell Culture

Human epidermal keratinocytes and melanocytes were sourced from Gibco (Thermo Fisher Scientific, Waltham, MA, USA). The keratinocytes were suspended in an EpiLife medium (Thermo Fisher Scientific) supplemented with bovine pituitary extract (BPE, 0.2%), recombinant human insulin-like growth factor-1 (rhIGF-1, 0.01 ug/mL), hydrocortisone (0.18 ug/mL), human epidermal growth factor (0.2 ng/mL), and bovine transferrin (BT, 5 ug/mL) (Thermo Fisher Scientific). The melanocytes were suspended in Medium 254 (Thermo Fisher Scientific) supplemented with BPE (0.2%), fetal bovine serum (0.5%), rhIGF-1 (0.01 ug/mL), hydrocortisone (0.18 ug/mL), basic fibroblast growth factor (3 ng/mL), BT (5 ug/mL), heparin (3 ug/mL), and phorbol 12-myristate 13-acetate (10 ng/m) (Thermo Fisher Scientific). For the experiments, keratinocytes and melanocytes from passages 3–6 and 10–20 were utilized. For the coculture of keratinocytes and melanocytes, keratinocytes were seeded at 2 × 10^5^ cells/well to six-well plates. Four hours later, 1 × 10^5^ melanocytes/well in 2 mL of EpiLife media were added to the keratinocytes. After 24 h, the EpiLife medium was replaced with the supplement-free medium, and GANT61 (5–10 μM; Sigma Aldrich, St. Louis, MO, USA) or CaCl_2_ (1 mM) treatment was administered by adding these agents to the cell culture for 48 h.

### 2.3. H_2_O_2_ Treatment or UVB Radiation

Primary cultured normal human keratinocytes were seeded at 1.5 × 10^5^ cells/well to six-well plates and incubated for 24 h. The cells in each six-well plate were subjected to different concentrations (50, 100, 200, and 500 mM) and durations (4, 24, and 48 h) of H_2_O_2_ or irradiated with 200 mJ of UVB either once or once daily for 3 consecutive days, employing a WL 20 W lamp emitting 305–314 nm with a peak of 311 nm (Royal Philips, Amsterdam, The Netherlands). Cell harvesting for NRF2 expression level evaluation was immediately conducted after the corresponding durations of H_2_O_2_ treatment or 2, 4, and 24 h after the final irradiation. The EpiLife medium was replaced with a supplement-free EpiLife medium or phosphate-buffered saline (PBS) during the H_2_O_2_ treatment or the irradiation, respectively.

### 2.4. Knockdown of IFT88, GLI1, and NRF2

Melanocytes and keratinocytes were seeded at 1.5 × 10^5^ cells/well to six-well plates and incubated for 24 h. The cells were transfected with 25 nM CRISPR-CAS9 sgRNA targeting human IFT88, GLI1, NRF2, or negative control sgRNA (Integrated DNA Technologies, San Diego, CA, USA) using the CRISPRMAX transfection reagent (Thermo Fisher Scientific). The cells were used for experiments 48 h after transfection. For the coculture of keratinocytes and melanocytes, 24 h later, 1 × 10^5^ melanocytes/well in 2 mL of EpiLife medium were added to transfected keratinocytes and incubated for another 24 h. Treatment with the SMO agonist (SAG; 1 µM; Sigma Aldrich) or Shh protein (100 nM; R&D Systems, Minneapolis, MN, USA) involved adding these substances to the transfected cells for 24 h after replacing the EpiLife medium with a supplement-free EpiLife medium. All collected cells were used for Western blot analysis, immunohistochemistry, confocal microscopy, tyrosinase activity assay, and melanin content assay.

### 2.5. Real-Time PCR Analysis

cDNA was synthesized from total RNA using a cDNA Synthesis Kit for RT-PCR (Promega, Fitchburg, WI, USA). The mRNA levels relative to GAPDH were measured using qPCR with a Light Cycler Real-Time PCR (Roche, Mannheim, Germany). The primer sequences used were as follows: IFT88 (NM 001353565) 5′-ATTGCCAATAGTTGTGGAGACTT-3′ (forward) and 5′-CTCGCTGTCTCACCAGGACT-3′ (reverse); PTCH1 (NM 001083605) 5′-TGGATGTCATGGCTTATCCAG-3′ (forward) and 5′-CATTAACTGGAACATGGTCTGC-3′ (reverse); GLI1 (NM_001160045) 5′-ATCAACTCGCGATGCACA-3′ (forward) and 5′-ATTCATCTGGGCTGGGAAT-3′ (reverse); GLI2 (NM_001374354) 5′-ACGGCACTGGATGACACAC-3′ (forward) and 5′-AGTGCTGGACACCTGGTTG-3′ (reverse); GLI3 (NM_000168) 5′-ACATGGAATATCTTCATGCTATGG-3′ (forward) and 5′-GGTGATATGGACAGTGTACGTTTT-3′ (reverse); and GAPDH (NM_001357943) 5′-TCCACTGGCGTCTTCACC-3′ (forward) and 5′-GCAGAGATGATGACCCTTT-3′ (reverse).

### 2.6. Western Blot Analysis

Equal amounts of extracted proteins (20 μg) were resolved via 8–12.5% sodium dodecyl sulfate-polyacrylamide gel electrophoresis and then transferred to nitrocellulose membranes. The membranes were probed with antibodies against various proteins, including IFT88 (rabbit polyclonal; Proteintech, Chicago, IL, USA), tyrosinase, PMEL17, GLI1, K14, K10, PAR2, heme oxygenase-1 (HO-1) (mouse monoclonal; Santa Cruz Biotechnology, Dallas, TX, USA), involucrin, PTCH1, GLI2 (goat; Santa Cruz Biotechnology), Shh (rabbit polyclonal; Cell Signaling Technology, Beverly, MA, USA), and NRF2 (rabbit polyclonal; Abcam, Cambridge, UK). The membranes were further incubated with anti-rabbit, anti-mouse, or anti-goat horseradish peroxidase-conjugated antibodies (Santa Cruz Biotechnology) and then treated with an enhanced chemiluminescence solution (Thermo, Rockford, IL, USA). Signals were captured using an image reader (LAS-3000; Fuji Photo Film, Tokyo, Japan). The membranes were re-probed with a mouse monoclonal anti-β-actin antibody (Sigma Aldrich) and processed as described above. The protein bands were analyzed via densitometry.

### 2.7. Immunohistochemistry and Confocal Microscopy

For the immunofluorescence staining of the biopsied skin specimens, the sections were pre-incubated with 3% bovine serum albumin after deparaffinization and rehydration. The sections were stained as follows: anti-NRF2 antibody (1:200 dilution), followed by Alexa-Fluor-labeled goat anti-rabbit IgG (1:200, 488; Molecular Probes, Eugene, OR, USA), anti-HO-1 antibodies (1:100) followed by Alexa-Fluor-labeled goat anti-mouse IgG (1:200, 594; Molecular Probes), or anti-involucrin antibodies (1:200) followed by Alexa-Fluor-labeled donkey anti-goat IgG (1:200, 594; Molecular Probes). For the staining of the cultured cells, the cells were fixed in 2% paraformaldehyde. The fixed cells were sequentially double-stained as follows: anti-NRF2 antibody followed by Alexa-Fluor-labeled goat anti-rabbit IgG and anti-ARL13 antibodies (1:200, mouse monoclonal: Santa Cruz Biotechnology), followed by Alexa-Fluor-labeled goat anti-mouse IgG and anti-acetylated-tubulin (1:500, mouse monoclonal; Sigma-Aldrich), followed by Alexa-Fluor-labeled goat anti-mouse IgG and anti-ARL13B antibodies (1:200, rabbit polyclonal; Proteintech), followed by Alexa-Fluor-labeled goat anti-rabbit IgG. The nuclei were counterstained with Hoechst 33258 (Sigma Aldrich). Fluorescence images were obtained and evaluated using an image analysis system (Dp Manager 2.1; Olympus Optical Co., Tokyo, Japan) and Wright Cell Imaging Facility ImageJ software version 1.54d (https://imagej.net/ij/download.html, accessed on 26 March 2023). Confocal microscopy images were obtained using EZ-C1 3.8 software (Nikon, Tokyo, Japan) and evaluated using NIS-Elements AR 3.2 (Nikon).

### 2.8. ROS Assay

ROS were detected using the Total ROS Detection Kit (Enzo Life, Frmingdale, NY, USA) according to the manufacturer’s instructions. Keratinocytes were seeded at 1.5 × 10^5^ cells/well to six-well plates and incubated for 24 h. An EpiLife medium was replaced with phosphate-buffered saline (PBS) during the irradiation. After irradiation with 200 mJ of UVB, the keratinocytes were incubated with a ROS detection solution for 1 h at 37 °C in the dark, according to the time schedule. The ROS levels were immediately measured using a fluorescence/multimode microplate reader (Spark; TECAN, Männedorf, Switzerland).

### 2.9. Tyrosinase Activity Assay

Tyrosinase activity was assayed in keratinocyte–melanocyte cocultures based on DOPA oxidase activity, using a modified version of the described method [19,20]. For coculture of keratinocytes and melanocytes, 1.5 × 10^5^ keratinocytes/well were transfected with indicated genes for 24 h and 1 × 10^5^ melanocytes/well were added to transfected keratinocytes and incubated for another 48 h. The cells were suspended and lysed in a phosphate buffer containing 1% Triton X-100. Cell-free extracts were obtained by centrifuging cell lysates at 10,000× *g* for 10 min. The protein concentrations of the supernatants were measured with Bradford assays and adjusted using the lysis buffer. We placed 90 μL of each lysate in a well of a 96-well plate and added 10 μL of L-DOPA. After incubation at 37 °C, absorbance was measured every 10 min for at least 1 h at 475 nm using a fluorescence/multimode microplate reader (Spark).

### 2.10. Melanin Content Assay

The melanin content of the keratinocyte–melanocyte cocultures was determined with minor modifications to the described method [21,22]. For the coculture of keratinocytes and melanocytes, 1.5 × 10^5^ keratinocytes/well were transfected with indicated genes for 24 h and 1 × 10^5^ melanocytes/well were added to the transfected keratinocytes and incubated for another 48 h. Briefly, after being washed with PBS, cell pellets were dissolved and solubilized with 1 N of NaOH at 80 °C for 2 h. After centrifugation at 12,000× *g* for 10 min, the absorbance of the supernatants was measured at a wavelength of 475 nm. To determine the actual melanin formation from the same number of cells, the total melanin content of each pellet was divided by the number of melanocytes.

### 2.11. Statistical Analysis

Statistical analyses were performed using GraphPad Prism 5 (GraphPad Software, La Jolla, CA, USA). The significance threshold was set at *p* < 0.05, with specific significance levels denoted as * *p* < 0.05, ** *p* < 0.01, and *** *p* < 0.001. Statistical comparisons between pairs of groups were performed using a two-tailed Student’s unpaired *t*-test (parametrical data). A one-way analysis of variance was used to compare multiple groups and parameters. Mean ± standard deviation (SD) values were calculated for the in vitro experimental data. For the human sample data, differences between the non-lesions and lesions were assessed using the Mann–Whitney U test and were expressed as the mean ± standard error of the mean.

## 3. Results

### 3.1. NRF2 Downregulation in the Lesional Epidermis of Patients with Melasma

**Figure 1:**
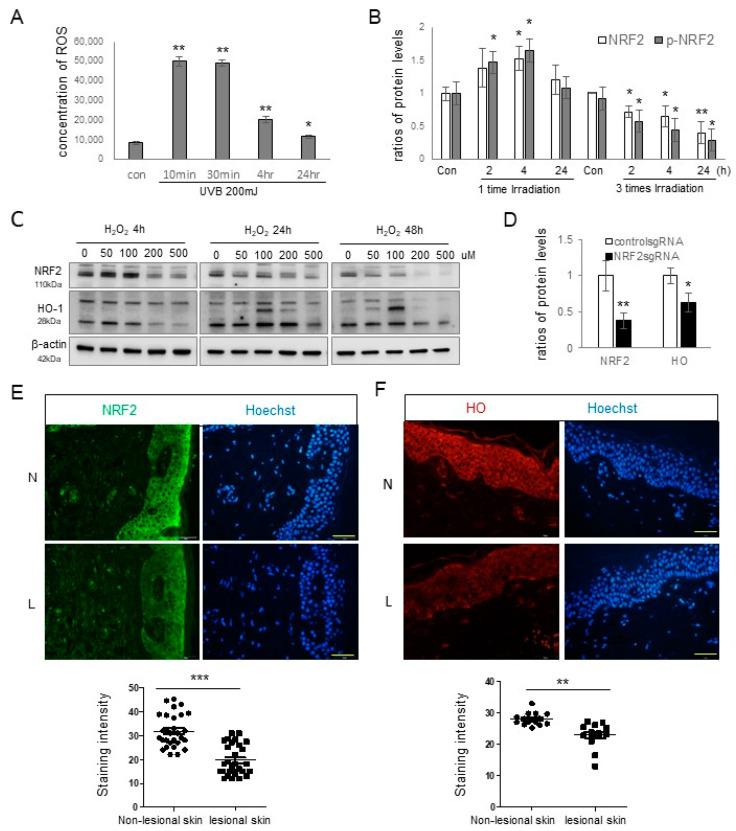
NRF2 downregulation in the lesional epidermis of patients with melasma. (**A**) Reactive oxygen species concentrations at various time points in primary cultured keratinocytes following UVB irradiation. (**B**) Western blot analyses showing NRF2 protein level ratios after single and repeated UVB radiation. (**C**) Western blot analyses illustrating NRF2 and HO-1 protein level ratios over time in primary cultured normal human keratinocytes treated with different concentrations of H_2_O_2_. (**D**) Western blot analyses presenting HO-1 protein level ratios in cultured human keratinocytes with or without *NRF2* knockdown. β-actin served as the internal control for the Western blot analysis. The data are presented as means ± SD from four or eight independent experiments. (**E**,**F**) Representative immunofluorescence staining using anti-NRF2 (**E**) and anti-HO-1 antibodies (**F**) in the lesional (L) and non-lesional (N) epidermis of patients with melasma. The nuclei were counterstained with Hoechst 33258 (scale bar = 0.05 mm), and the intensities were quantified using ImageJ software 1.54d. * *p* < 0.05, ** *p* < 0.01, *** *p* < 0.001.

Oxidative stress has been proposed as a contributing factor in the pathogenesis of melasma [4,5,6]. Consequently, the presence of oxidative stress in melasma was explored by assessing the expressions of NRF2 and one of its targets, HO-1, in keratinocytes. Notably, one of the primary instigators of ROS production and oxidative stress is UV radiation [23,24]. A single exposure to UVB resulted in increased intracellular ROS levels (Figure 1A) and elevated NRF2 protein expression (Figure 1B and Figure S1). However, after three consecutive days of UVB exposure, the NRF2 expression levels declined (Figure 1B). Elevated ROS levels can trigger NRF2 upregulation. As such, we investigated the expressions of NRF2 and HO-1 proteins in primary cultured keratinocytes following treatment with H_2_O_2_. Their levels exhibited time- and dose-dependent increases, up to 48 h and 100 μM (Figure 1C). Additionally, *NRF2* knockdown in primary cultured keratinocytes led to reduced HO-1 expression (Figure 1D and Figure S1). Immunofluorescence staining was conducted using anti-NRF2 and anti-HO-1 antibodies in 16 patients, and seven of them had results, suggesting that the relative expression levels of NRF2 (Figure 1E) and HO-1 (Figure 1F) were diminished in the lesional epidermis compared with the non-lesional epidermis.

### 3.2. IFT88 and Hh Signaling Molecules Involved in Ciliogenesis Were Reduced by NRF2 Downregulation

**Figure 2:**
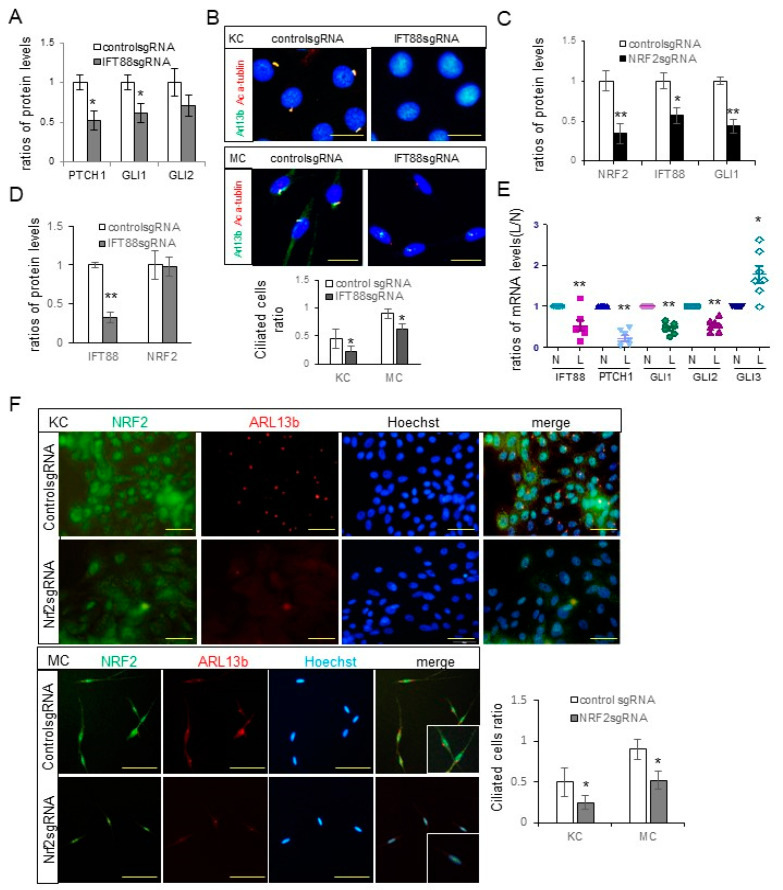
Downregulation of NRF2 led to reduced expressions of IFT88 and Hh signaling molecules involved in ciliogenesis. (**A**) Western blot analyses depicting the ratios of PTCH, GLI1, and GLI2 levels in cultured keratinocytes subjected to *IFT88* knockdown. (**B**) Confocal microscopy images illustrating primary cilia stained with anti-acetylated α-tubulin (Ac α-tubulin) and/or ARL13b antibodies in cultured human keratinocytes and melanocytes, with or without *IFT88* knockdown (bar = 0.05 mm). The ciliated cell ratios were calculated by counting the number of ciliated cells among 30 cells. (**C**,**D**) Western blot analyses showing the ratios of NRF2, IFT88, and/or GLI1 levels in cultured keratinocytes with knockdowns of *NRF2* (**C**) or *IFT88* (**D**). (**E**) Real-time PCR results displaying the ratios of IFT88, PTCH1, and GLI1-3 mRNA levels in lesional compared to non-lesional skin specimens (seven sets) from melasma patients with downregulated NRF2. (**F**) Representative immunofluorescence staining for primary cilia using anti-NRF2 and anti-ARL13b antibodies in primary cultured human keratinocytes and melanocytes with or without *NRF2* knockdown (scale bar = 0.05 mm). β-actin and GAPDH served as internal controls for the Western blot analysis and real-time PCR, respectively. The data are presented as means ± SD from four independent experiments. * *p* < 0.05, ** *p* < 0.01.

The downregulation of NRF2 in melasma skin lesions (Figure 1E) is associated with the expected occurrence of oxidative stress. Given that oxidative stress can disrupt ciliogenesis [7,8], the impact of NRF2 downregulation on primary cilia was examined, after confirming the role of IFT in keratinocyte and melanocyte ciliogenesis [13]. Knocking down *IFT88* in keratinocytes led to the downregulation of PTCH, GLI1, and GLI2 (Figure 2A and Figure S2). Additionally, confocal microscopy demonstrated that *IFT88* knockdown reduced primary cilia that were positive for acetylated α-tubulin or both ARL13b and acetylated α-tubulin in cultured keratinocytes and melanocytes (Figure 2B). *NRF2* knockdown resulted in reduced expressions of IFT88 and GLI1 in cultured keratinocytes (Figure 2C and Figure S2). However, *IFT88* knockdown did not affect the expression levels of NRF2 (Figure 2D and Figure S2). Real-time PCR performed on samples from seven patients with NRF2 downregulation showed that the relative ratios of IFT88, GLI1, GLI2, and PTCH mRNAs in lesional to non-lesional skin specimens decreased to almost half or less, whereas those of GLI3 increased (Figure 2E). Furthermore, immunofluorescence staining indicated that *NRF2* knockdown reduced the numbers of ARL13b-positive primary cilia in cultured keratinocytes and melanocytes (Figure 2F).

### 3.3. Melanin Pigmentation Was Enhanced by NRF2 Knockdown via IFT88 and GLI1

**Figure 3:**
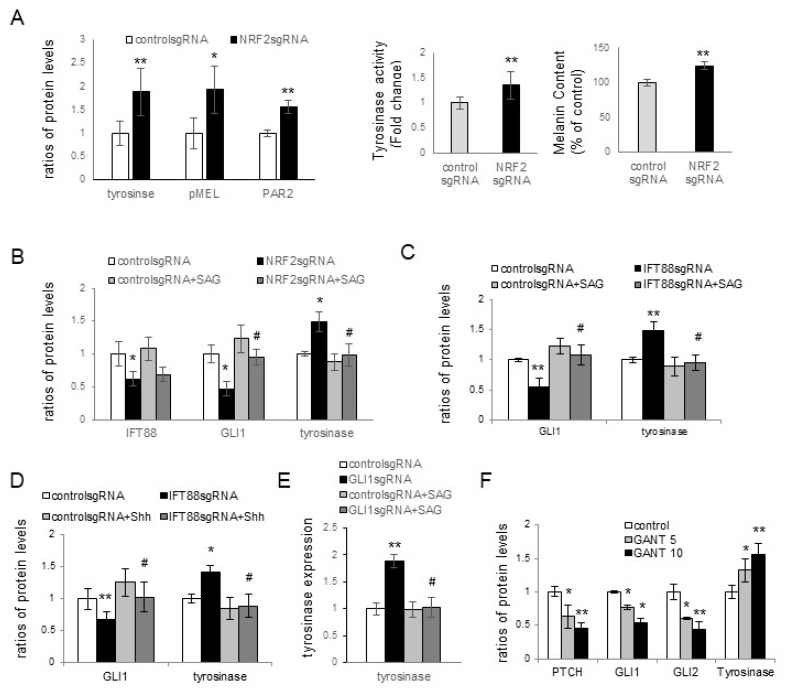
Enhancement of melanin pigmentation via *NRF2* knockdown involving IFT88 and GLI1. (**A**) Western blot analyses depicting varying levels of tyrosinase, PMEL, and PAR2, and assays showing tyrosinase activity and melanin contents in cultured keratinocytes with *NRF2* knockdown. (**B**–**E**) Western blot analyses revealing different ratios of NRF2, IFT88, GLI1, and/or tyrosinase levels in cultured keratinocytes with *NRF2* knockdown in the absence and presence of SAG (**B**), *IFT88* knockdown in the absence and presence of SAG (**C**), *IFT88* knockdown in the absence and presence of Shh 200 (**D**), and *GLI1* knockdown in the absence and presence of SAG (**E**). (**F**) Western blot analyses presenting different ratios of PTCH1, GLI1, GLI2, and tyrosinase levels in cultured keratinocyte–melanocyte cocultures treated with or without GANT61. β-actin served as an internal control. The data represent the means ± SD from four independent experiments. * *p* < 0.05, ** *p* < 0.01 vs. control sgRNA, # *p* < 0.05 vs. without SAG treatment.

NRF2, downregulated in melasma lesional skin (Figure 1E), played a role in ciliogenesis regulation through IFT88 and Hh signaling molecules (Figure 2C–F). Consequently, the impact of NRF2, IFT88, and GLI1 in hyperpigmentation was assessed, both in the presence or absence of Hh signaling activation using the smoothened agonist 1.1 (SAG) or Shh protein. *NRF2* knockdown resulted in increased expressions of tyrosinase, the premelanosomal protein (PMEL17), and protease-activated receptor-2 (PAR2) in keratinocytes cocultured with normal melanocytes (Figure 3A). Furthermore, *NRF2* knockdown in these cocultures led to elevated tyrosinase activity and melanin contents (Figure 3A and Figure S3). The levels of tyrosinase and PAR2, which were increased due to *NRF2* knockdown, were restored by SAG treatment. However, the SAG did not restore downregulated NRF2 (Figure 3B and Figure S3). *IFT88* knockdown in keratinocytes also upregulated tyrosinase. The SAG (Figure 3C and Figure S3) and Shh protein (Figure 3D and Figure S3) restored the tyrosinase levels following *IFT88* knockdown without affecting the IFT88 expression levels. GLI1 downregulation, achieved either through *GLI1* knockdown (Figure 3E and Figure S3) or via treatment with GANT61, a GLI1 inhibitor (Figure 3F and Figure S3), resulted in increased tyrosinase protein levels. The SAG reversed GLI1 downregulation but also downregulated tyrosinase (Figure 3E).

### 3.4. NRF2, IFT88, and GLI1 Knockdown Promoted Keratinocyte Differentiation and Consequent Hyperpigmentation

**Figure 4:**
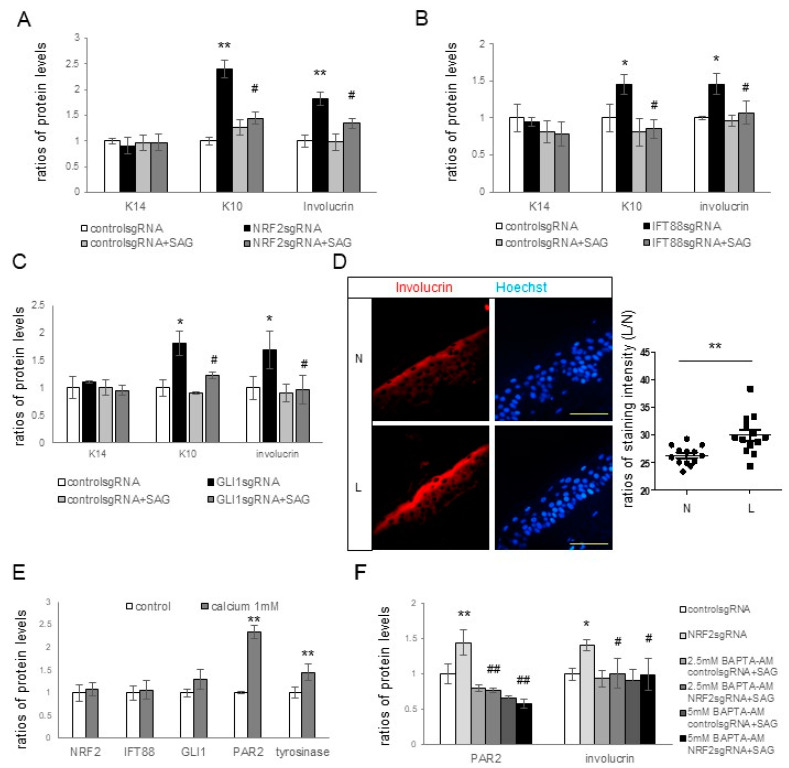
Effects of *NRF2*, *IFT88*, and *GLI1* knockdowns on keratinocyte differentiation and subsequent hyperpigmentation. (**A**–**C**) Western blot analyses illustrating the relative levels of K14, K10, and involucrin in cultured keratinocytes with or without knockdowns of *NRF2* (**A**), *IFT88* (**B**), or *GLI1* (**C**) in the absence and presence of SAG. (**D**) Representative immunofluorescence staining using anti-involucrin antibodies (**B**) in the lesional (L) and non-lesional (N) epidermis of seven patients with melasma. The nuclei were counterstained with Hoechst 33258 (bar = 0.05 mm), and the intensities were measured using ImageJ software 1.54d. (**E**,**F**) Western blot analyses for the ratios of tyrosinase levels in keratinocyte–melanocyte cocultures and the ratios of PAR2, K10, involucrin, NRF2, IFT88, and/or GLI1 levels in cultured keratinocytes, including those treated with calcium (**E**) and keratinocytes with or without NRF2 knockdown in the absence and presence of Bapta-AM (**F**). β-actin was used as an internal control for the Western blot analysis. The data are presented as the means ± SD from four independent experiments. * *p* < 0.05, ** *p* < 0.01 vs. control sgRNA; # *p* < 0.05, ## *p* < 0.01 vs. without SAG treatment.

Our findings suggested that NRF2 regulates ciliogenesis and melanin pigmentation through IFT88 and GLI1. The primary cilia and Hh signaling pathway play crucial roles in epidermal homeostasis, including keratinocyte proliferation and differentiation [25,26,27,28], ultimately affecting skin pigmentation [29]. We delved into the roles of NRF2, IFT88, and GLI1 in keratinocyte proliferation and differentiation and their connections to pigmentation changes. *NRF2* knockdown resulted in increased expressions of K10 and involucrin, which could be restored with SAG treatment (Figure 4A). However, K14 expression remained unaffected (Figure 4A and Figure S4). The knockdown of *IFT88* (Figure 4B and Figure S4) or *GLI1* (Figure 4C and Figure S4) led to the upregulation of K10 and involucrin proteins without affecting K14. These changes could be reversed with SAG treatment. Immunofluorescence staining unveiled a higher involucrin expression in the lesional epidermis of the seven melasma patients with NRF2 downregulation (Figure 4D and Figure S4). Keratinocytes cultured under high calcium levels displayed enhanced expressions of K10, involucrin, tyrosinase, and PAR2 (Figure 4E and Figure S4). Notably, high calcium levels had no impact on the expressions of NRF2, IFT88, or GLI1 in keratinocytes (Figure 4E). The increased involucrin and PAR2 protein levels after *NRF2* knockdown were restored with Bapta-AM, a calcium chelator, without affecting the NRF2 expression levels (Figure 4F and Figure S4).

## 4. Discussion

NRF2 is a central regulator of cellular antioxidant defenses. Under conditions of oxidative stress, NRF2 relocates to the cell nucleus and activates the expressions of a wide array of target genes, including HO-1 [30]. The diminished levels of HO-1 proteins following *NRF2* knockdown (Figure 1C) confirmed HO-1 as a primary target of NRF2 within keratinocytes. UV radiation, a known source of ROS [2], elevated ROS levels and NRF2 protein expression in keratinocytes after a single UVB exposure (Figure 1A,B). In response to H_2_O_2_-induced oxidative stress, the relative expressions of NRF2 and HO-1 in keratinocytes were increased (Figure 1C), reflecting an antioxidant response aimed at combating oxidative stress. However, repeated UVB irradiation downregulated NRF2 (Figure 1B), suggesting that oxidative stress was hardly overcome under repeated UVB irradiation. Given that chronic UV exposure is a significant contributor to melasma development [1], it is reasonable to expect NRF2 and HO-1 downregulation in the lesional epidermis of melasma patients (Figure 1E,F). Nevertheless, melasma development is also associated with factors unrelated to UV exposure [1]. In addition to UV radiation, ROS can be generated by endogenous processes such as cellular metabolism and exogenous factors other than UV radiation [31]. While the role of UV-independent melasma triggers in oxidative stress requires further investigation, the results from the H_2_O_2_ treatment suggested that decreased cellular antioxidant capacity may cause oxidative stress in melasma, regardless of its association with UV.

The downregulation of Hh signaling molecules, including PTCH, GLI1, and GLI2 (Figure 2A), and a decrease in the number of ciliated cells (Figure 2B) following *IFT88* knockdown, verified the role of IFT88 in ciliogenesis in keratinocytes and melanocytes. The reduced expression levels of IFT88 and GLI1 (Figure 2C) and number of ciliated cells (Figure 2F) following *NRF2* knockdown, with no reciprocal impacts on NRF2 expression following *IFT88* knockdown (Figure 2D), suggested that NRF2 can regulate ciliogenesis via IFT88 and GLI1. The downregulation of IFT88 and GLI1 in the lesional skin of patients with melasma with NRF2 downregulation (Figure 2E) substantiates the role of oxidative stress in ciliogenesis in melasma. NRF2 has been established as a pivotal regulator of ciliogenesis [32,33], with various genes related to ciliogenesis and the Hh signaling pathway identified as NRF2 target genes [32].

Melasma is a skin disorder marked by hyperpigmentation, which indicates that oxidative-stress-induced inhibition of ciliogenesis contributes to hyperpigmentation. Skin pigmentation is intricately linked to melanin synthesis, the transfer of melanosomes to keratinocytes, and melanosome degradation. Increased tyrosinase activity indicated an upregulation in melanin synthesis with *NRF2* knockdown in keratinocyte–melanocyte cocultures (Figure 3A). However, other factors, such as stimulated melanosome transfer to keratinocytes and reduced melanosome degradation, may have contributed to the elevation of melanin contents. The role of PAR2 in the transfer of melanosomes to keratinocytes is well known [29,34]. The upregulation of tyrosinase, PMEL17, and PAR-2 in keratinocytes with downregulated NRF2, IFT88, or GLI1 (Figure 3A–E) indicated that the inhibition of oxidative stress-related ciliogenesis enhanced melanin synthesis and melanosome transfer. The restoration of the increased levels of tyrosinase or PAR-2 with GLI1, but not NRF2 or IFT88, with SAG or Shh 200 treatments (Figure 3B–E) suggested that GLI1 is a downstream molecule for the inhibitory role of primary cilia in hyperpigmentation. The elevation of tyrosinase expression with GANT61, a GLI1 inhibitor (Figure 3F), supported the results obtained with GLI1.

However, the mechanism by which primary cilia suppress melanin synthesis and melanosome transfer remains unclear. Primary cilia and the Hh signaling pathway are known to contribute to epidermal homeostasis, including keratinocyte proliferation or differentiation [26,27,28]. Consequently, markers for keratinocyte differentiation and proliferation, as well as those of tyrosinase and PAR2, were examined in the presence and absence of *NRF2*, *IFT88*, or *GLI1* knockdown. The expression levels of involucrin and K10, but not of K14, in keratinocytes increased upon the knockdown of *IFT88* or *GLI1* as well as *NRF2* (Figure 4A–C), suggesting an inhibitory role of primary cilia in keratinocyte differentiation, but not in keratinocyte proliferation. The restoration of upregulated K10 and involucrin in cultured keratinocytes with *NRF2*, *IFT88*, or *GLI1* knockdown with the SAG (Figure 4A–C) supported the findings that primary cilia are involved in the inhibition of keratinocyte differentiation. Enhanced keratinocyte differentiation was also detected in the lesional skin of melasma patients with downregulated IFT88 and GLI1 (Figure 4D). Calcium upregulated involucrin, tyrosinase, and PAR2 (Figure 4E), whereas calcium chelator restored the upregulation of involucrin and PAR2 with *NRF2* knockdown (Figure 4F). However, neither calcium nor calcium chelators altered the expression of NRF2, IFT88, or GLI1 (Figure 4E,F). Although more studies are necessary to reach definitive conclusions, the connection from NRF2 downregulation to IFT88 and GLI1 downregulation may be involved in increasing melanin synthesis and melanosome transfer by stimulating keratinocyte differentiation in melasma.

## 5. Conclusions

**Figure 5:**
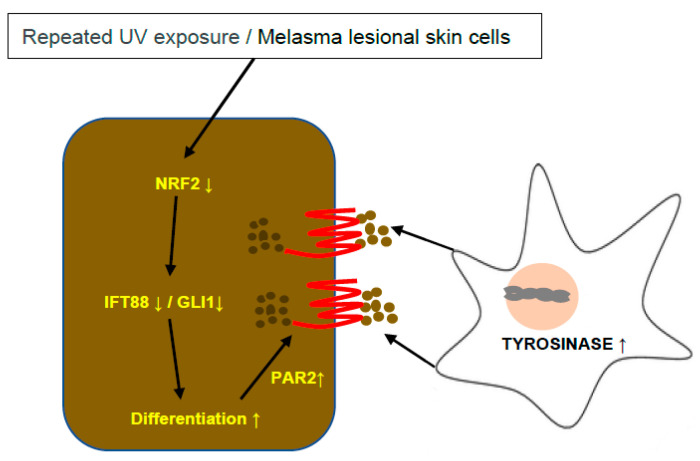
Schematic view of the role of *NRF2*-knockdown-induced ciliogenesis inhibition in skin hyperpigmentation. NRF2 downregulation caused by repeated UV exposure or melasma inhibited ciliogenesis and Hh signaling molecules, such as IFT88 and GLI1, stimulating keratinocyte differentiation with melanin synthesis and melanosome transfer to the keratinocytes, which resulted in skin hyperpigmentation.

Overall, oxidative stress in melasma inhibited ciliogenesis and Hh signaling in lesional keratinocytes with NRF2 downregulation, which then stimulated keratinocyte differentiation with melanin synthesis and melanosome transfer to the keratinocytes, resulting in skin hyperpigmentation (Figure 5).

## 6. Patents

Patent information is included in the Materials and Methods section of the original manuscript.

## Data Availability

Data is contained within the article or Supplementary Material.

## Supplementary Materials

The following supporting information can be downloaded at: https://www.mdpi.com/article/10.3390/antiox12111969/s1, Figure S1: Bands from Western blot analyses for Figure 1B (A) and Figure 1D (B). Figure S2: Bands from Western blot analyses for Figure 2A (A), Figure 2C (B) and Figure 2D (C). Figure S3: Bands from Western blot analyses for Figure 3A (A), Figure 3B (B), Figure 3C (C), Figure 3D (D), Figure 3E (E) and Figure 3F (F). Figure S4: Bands from Western blot analyses for Figure 4A (A), Figure 4B (B), Figure 4C (C), Figure 4E (D) and Figure 4F (E).
