# Supplementary material for: Oxidative Stress Induces Skin Pigmentation in Melasma by Inhibiting Hedgehog Signaling
Source: Antioxidants (Basel). 2023 Nov 6;12(11):1969. doi: 10.3390/antiox12111969 (PMC10669456; doi:10.3390/antiox12111969)
Supplement: Supplementary file 1 [file antioxidants-12-01969-s001.zip › antioxidants-2584070-supplementary.pdf]

Oxidative Stress Induces Skin Pigmentation in Melasma by Inhibiting Hedgehog Signaling

Nan-Hyung Kim and Ai-Young Lee *

Department of Dermatology, Dongguk University Ilsan Hospital, 814 Siksa-dong, Ilsandong-gu, Goyang-si 410-773, Gyeonggi-do, Republic of Korea

* Correspondence: leeay@dumc.or.kr; Tel.: +82-319617250

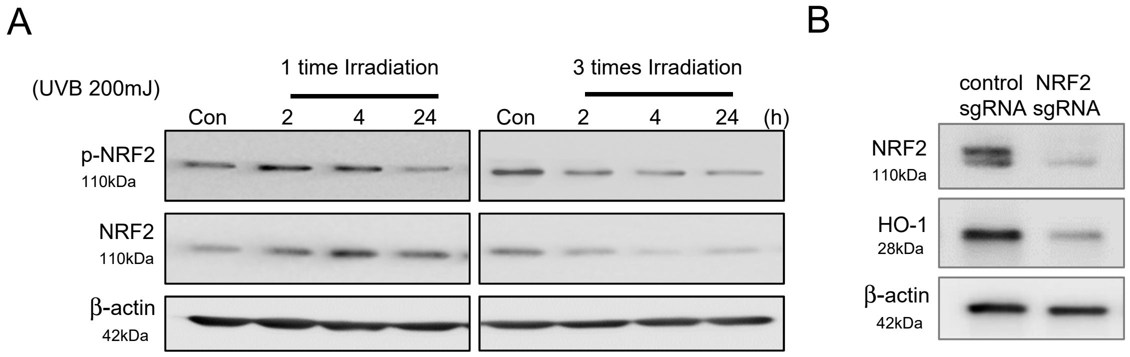

Figure S1. Bands from western blot analyses for Figure 1B (A) and 1D (B)

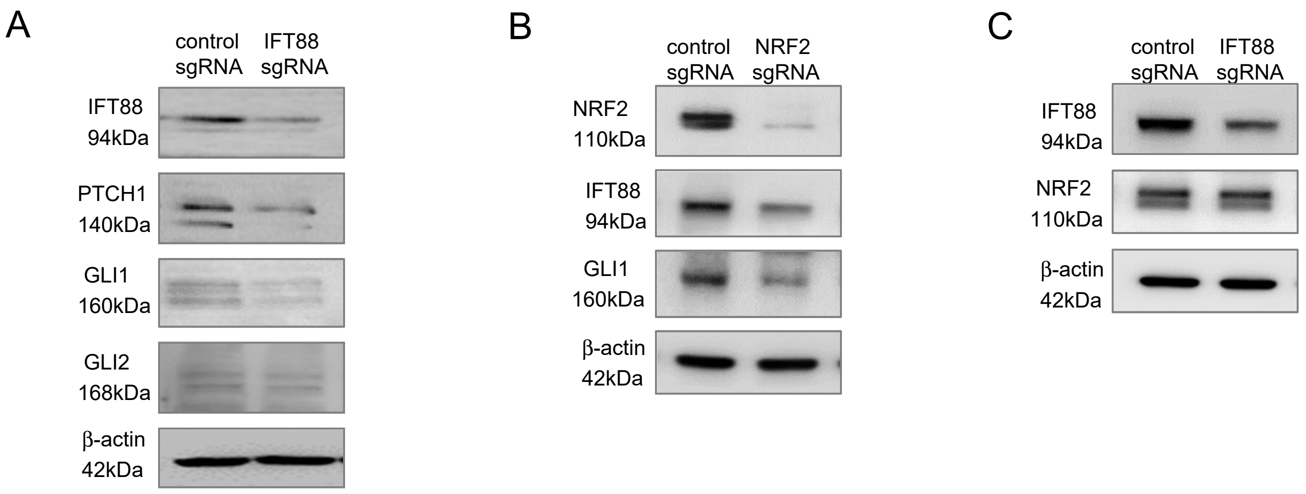

Figure S2. Bands from western blot analyses for Figure 2A (A), 2C (B), and 2D (C).

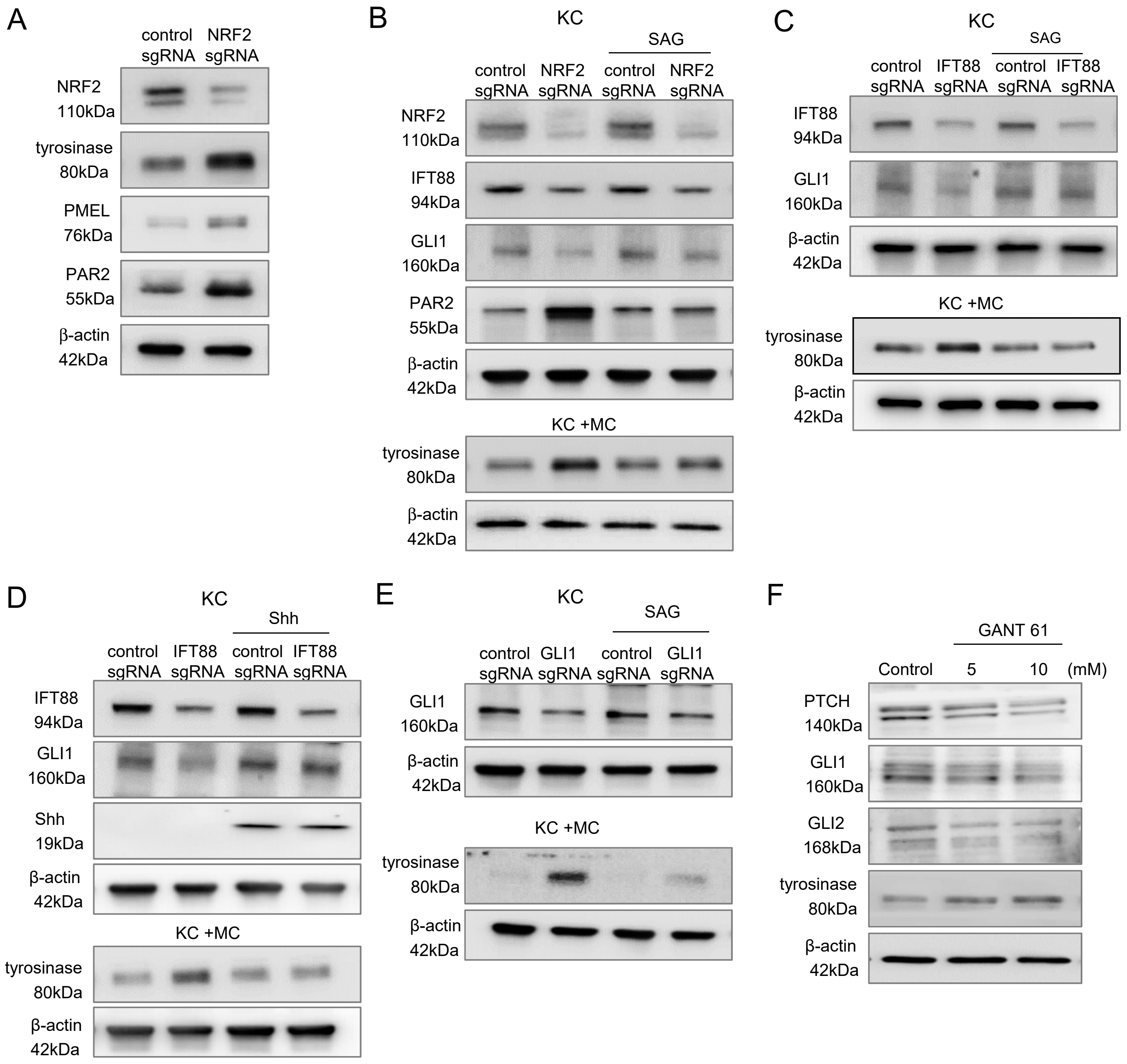

Figure S3. Bands from western blot analyses for Figure 3A (A), 3B (B), 3C (C), 3D (D), 3E (E), and 3F (F)

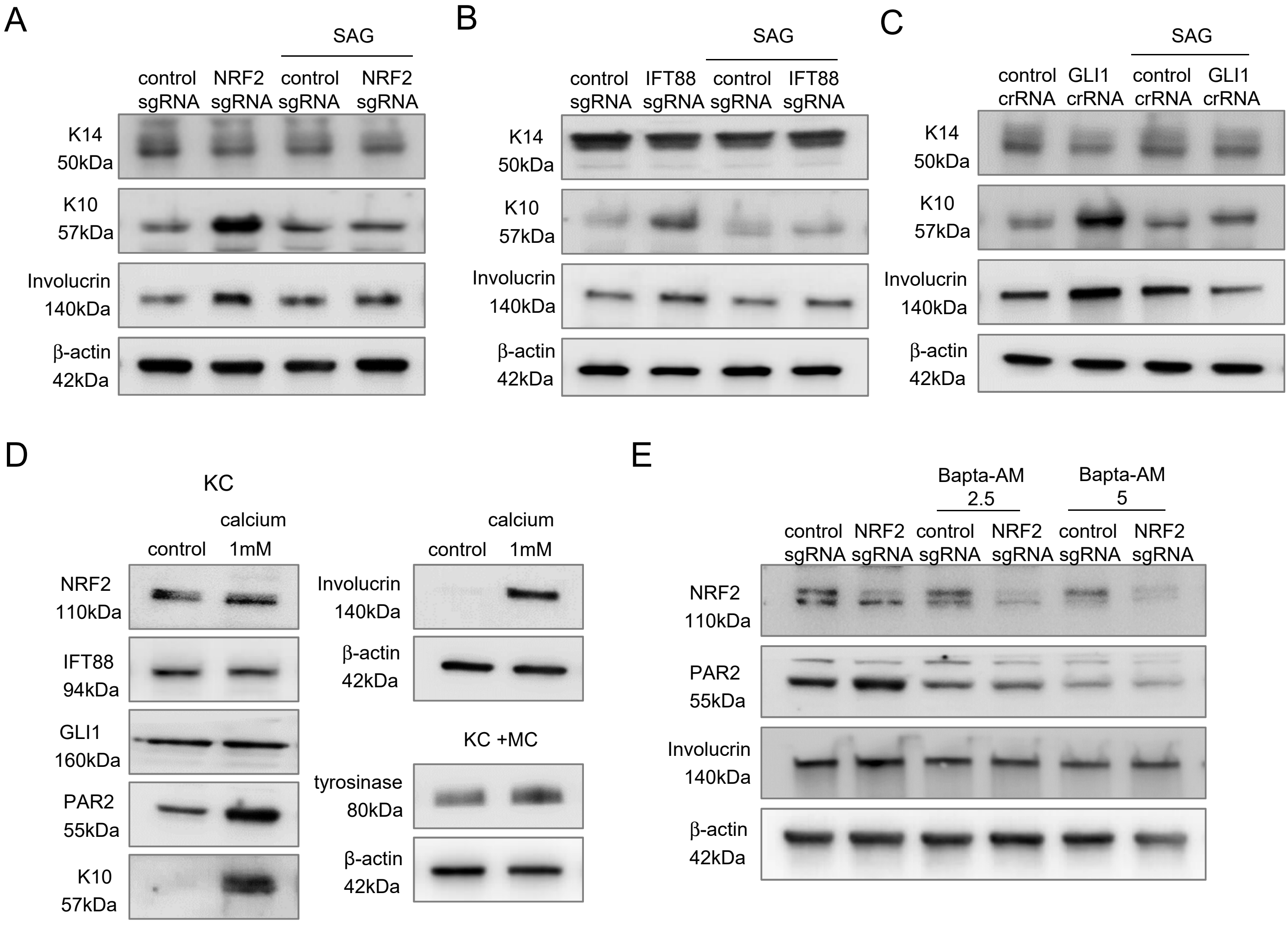

Figure S4. Bands from western blot analyses for Figure 4A (A), 4B (B), 4C (C), 4E (D), and 4F (E)
